# Informationsweitergabe zur COVID-19-Impfung: Quantitative Online-Befragung zu Akteuren, Inhalten, Quellen und Motiven

**DOI:** 10.1007/s00103-023-03677-5

**Published:** 2023-03-15

**Authors:** Paula Memenga, Jule Scheper, Elena Link

**Affiliations:** Institut für Journalistik und Kommunikationsforschung, Hochschule für Musik, Theater und Medien Hannover, Expo Plaza 12, 30539 Hannover, Deutschland

**Keywords:** Meinung und Erfahrung, Informationshandeln, Informationssuche, Online-Informationsquellen, Soziales Umfeld, Opinion and experience, Information-seeking behavior, Information seeking, Online information sources, Social environment

## Abstract

**Hintergrund:**

Welche Meinung eine Person zur COVID-19-Impfung vertritt und wie ihre Impfentscheidung ausfällt, hängt u. a. von den sie erreichenden Informationen ab. Es scheint somit zentral, das Phänomen der Informationsweitergabe im Kontext der COVID-19-Impfung zu untersuchen. Die Studie exploriert, wer welche Informationen zur Impfung an wen weitergibt, wie die Weitergabe mit der Nutzung von Informationsquellen zusammenhängt und welche Motive bestehen.

**Methode:**

Im Zeitraum vom 19.06. bis 13.07.2021 wurde eine Umfrage mit 833 Personen über das deutsche nicht-repräsentative Social Sciences’ (SoSci) Panel durchgeführt. Erfragt wurden die Informationsweitergabe an Fremde und Personen aus dem sozialen Umfeld, Inhalte und Motive sowie die Informationsquellen. Die Antworten von 828 Personen wurden mittels deskriptiver Statistiken und multipler linearer Regressionsanalysen ausgewertet.

**Ergebnisse:**

Informationen zur COVID-19-Impfung wurden tendenziell häufiger von älteren Personen, Frauen und Ungeimpften weitergegeben und umfassten Vorteile und Risiken der Impfung sowie persönliche Erfahrungen. Die Informationsweitergabe erfolgte häufiger im sozialen Umfeld als an Fremde. Personen, die sich über die Webseiten von Gesundheitsbehörden informieren, gaben häufiger Informationen zu Vorteilen sowie persönliche Erfahrungen weiter, während Personen, die YouTube oder Messenger-Dienste nutzen, häufiger Informationen zu Risiken teilten. Motive der Informationsweitergabe sind, *anderen Menschen helfen, sozialer Austausch, Aufmerksamkeit schaffen, Statusgewinn* und die *Angst vor Angriffen* oder *Verurteilung*.

**Diskussion:**

Die Informationsweitergabe zur COVID-19-Impfung ist inhaltlich wie motivational vielschichtig. Um die Impfbereitschaft zu fördern, sollte das Informationsangebot offizieller Webseiten ausgebaut sowie die Gesundheitskompetenz der Bevölkerung gestärkt werden.

## Abstract (en)

**Background:**

A person’s opinion about COVID-19 vaccination and their decision for or against the vaccination are determined, among other things, by the information they receive. Therefore, it seems crucial to examine the phenomenon of information sharing in the context of COVID-19 vaccination. The study explores who shares which information about vaccinations with whom, how the sharing of information is related to the seeking of information from various sources, and what the motives are.

**Method:**

From 19 June to 13 July 2021, we conducted an online survey with 833 people using the German non-representative Social Sciences’ (SoSci) panel. The questionnaire assessed information sharing with strangers and the social environment, the content, and the motives as well as the sources of information. The responses of 828 people were evaluated using descriptive statistics and multiple linear regression analyses.

**Results:**

Older people, women, and unvaccinated individuals were more likely to share information about COVID-19 vaccination. Information was more often shared in the social environment than with strangers. People shared information about benefits and risks of the vaccination as well as their own experiences with vaccinations. People who research websites of health authorities were more likely to share information about the benefits of vaccinations and their own experiences, while people using YouTube or messenger services were more likely to share information about risks. The motives for sharing information are to help others, social exchange, to raise awareness, to gain status, and the fear of being attacked or judged.

**Conclusion:**

Information sharing on COVID-19 vaccination is complex in terms of content and motivation. In order to promote vaccination readiness, the range of information offered by public health departments should be expanded and the population’s health literacy should be strengthened.

## Hintergrund

Impfungen sind eine der effektivsten Maßnahmen zur Prävention von Krankheiten und retten jährlich mehrere Millionen Menschenleben [1]. Einige Menschen zögern jedoch oder weigern sich, sich impfen zu lassen. Die sogenannte Impfmüdigkeit (*Vaccine Hesitancy*) gilt als eine der größten Bedrohungen für die globale Gesundheit [2] und ist auch bei der Bewältigung der COVID-19-Pandemie eine zentrale Herausforderung [3]. Die Entscheidung für oder gegen eine Impfung ist komplex und wird maßgeblich durch die Informationen beeinflusst, die eine Person erreichen und denen sich eine Person zuwendet [4–7]. Das Informationshandeln ist somit von zentraler Bedeutung für die individuelle Entscheidung für oder gegen eine Impfung und beeinflusst im Kontext der COVID-19-Impfentscheidung sogar möglicherweise den Pandemieverlauf.

Im Vergleich zu klassischen Formen des Informationshandelns, wie der aktiven Suche nach Informationen [8, 9], hat die sogenannte soziale Informationsweitergabe bislang eher wenig Beachtung in der kommunikationswissenschaftlichen Forschung gefunden. Individuen geben sowohl online als auch offline täglich Informationen wie persönliche Erfahrungen, Meinungen, Wissen und Beiträge aus anderen Quellen [10–13] weiter. Das Ergebnis der Informationsweitergabe kann als ein kollektives Gut verstanden werden, das andere in ihrem Wissen, ihrer Meinungsbildung und Entscheidungsfindung beeinflusst [14] und je nach Wahrheitsgehalt der Informationen nutzen oder schaden kann [13]. Vor allem soziale Medien bieten viele Möglichkeiten, zu jeder Zeit und von überall aus Informationen mit einzelnen Personen oder einem breiteren Publikum zu teilen – z. B. durch das *Posten *oder* Reposten *von Texten, Bildern, Videos oder Links [15]. Dadurch werden jedoch auch falsche und irreführende Informationen schnell verbreitet [16], die Verunsicherung und gesundheitsschädigendes Verhalten bedingen [17] sowie zu einem unüberschaubaren Informationsangebot beitragen können. Dieses Phänomen wird von der Weltgesundheitsorganisation (WHO) als *Infodemie* bezeichnet [18].

Welche Meinung jemand zu einer Impfung vertritt und wie die Impfentscheidung ausfällt, hängt unter anderem also davon ab, welche Informationen an diese Person weitergegeben werden [19]. Aus kommunikationswissenschaftlicher Sicht erscheint es daher zentral, das Phänomen der Informationsweitergabe im Kontext der COVID-19-Impfung genauer zu untersuchen. Bislang wurde die Informationsweitergabe überwiegend im Rahmen der Organisationsforschung [20–23] und erst im Zuge der zunehmenden Interaktionsmöglichkeiten im Internet auch in ersten Studien im gesellschaftlichen Kontext fokussiert [13, 14, 21, 24]. Aktuelle Studien, die auch das Informationsverhalten zur COVID-19-Impfung untersuchen, fokussieren sich dagegen meist auf die Informationssuche [25–27]. Die vorliegende Studie setzt hier mit der Untersuchung der Informationsweitergabe zur COVID-19-Impfung an und trägt zur weiteren Exploration des Phänomens bei.

Bislang ist wenig über die Akteure der Informationsweitergabe bekannt. Insbesondere vor dem Hintergrund der COVID-19-Pandemie, in der bedingt durch eine hohe Unsicherheit viele unterschiedliche Meinungen und Wahrnehmungen aufeinandertreffen, ist es wichtig zu verstehen, wer Informationen an wen weitergibt. Hier stellt sich die Frage, inwiefern spezifische Charakteristika wie Alter, Geschlecht oder der Impfstatus die Weitergabe bedingen. Alter und Geschlecht sind bekannte Determinanten des Gesundheitsinformationshandelns. So sind z. B. Personen, die nach Gesundheitsinformationen suchen, meist weiblich und jünger [27–29]. Es ist unklar, inwiefern diese Zusammenhänge auch hinsichtlich der COVID-19-Impfung bestehen.

Darüber hinaus können Informationen an unterschiedliche Empfänger*innen weitergegeben werden. Informationen, die an Personen aus dem sozialen Umfeld weitergegeben werden, wirken sich vermutlich stärker auf Meinungsbildung und Entscheidungsfindung aus, da das soziale Umfeld im Vergleich zu fremden Personen ein stärkeres Einflusspotenzial aufweist [30]. Das soziale Umfeld besteht jedoch häufig aus ähnlich denkenden Personen, während die Weitergabe an Fremde besonders mit der Möglichkeit einhergeht, auch andersdenkende Menschen zu erreichen und zu beeinflussen [31].

Des Weiteren ist bislang wenig darüber bekannt, *welche* Informationen weitergegeben werden und aus welchen Quellen diese stammen. Informationen zu den Risiken der COVID-19-Impfung oder negative Impferfahrungen wirken sich vermutlich auch negativ auf die Impfentscheidung aus [3], während Informationen zu den Vorteilen der Impfung eine Entscheidung für die Impfung fördern könnten [19]. So sind z. B. Sicherheitsbedenken negativ [19, 32] und die Annahme, dass die Impfung einen persönlichen Schutz vor Ansteckung bietet, positiv mit der Impfbereitschaft assoziiert [19, 25].

Die Motive der Informationsweitergabe sind vielfältig und spiegeln wichtige soziale und kommunikative Funktionen wider [15]. Die Informationsweitergabe kann bspw. in der intrinsischen Motivation begründet sein, anderen zu helfen [10, 33, 34], soziale Beziehungen aufzubauen und aufrechtzuerhalten [11, 33] oder Aufmerksamkeit für ein Thema zu schaffen [35]. Ebenso bedeutsam können auch extrinsische Motive wie Reputation und Status sein [11, 36]. Die Befürchtung, von anderen Personen abgelehnt, verurteilt oder angegriffen zu werden, kann hingegen eine Barriere für die Informationsweitergabe darstellen [37].

Die vorliegende Studie verfolgt das Ziel, detailliertere Einblicke in die Informationsweitergabe zur COVID-19-Impfung in Deutschland zu geben. Zunächst wird mit Blick auf Alter, Geschlecht und Impfstatus exploriert, wer Informationen zur COVID-19-Impfung an wen (soziales Umfeld, Fremde) weitergibt (F1). Anschließend werden die Inhalte der weitergegebenen Informationen analysiert (F2) und untersucht, inwiefern die Informationssuche über unterschiedliche Informationsquellen mit der Weitergabe dieser Informationen zusammenhängt (F3). Zuletzt werden Motive der Informationsweitergabe fokussiert (F4).

## Methode

Zur Beantwortung der Forschungsfragen (F1 bis F4) wurde eine Online-Befragung über das deutsche nicht-repräsentative Social Sciences’ (SoSci) Panel durchgeführt. Es handelt sich dabei um ein nicht-kommerzielles Panel für wissenschaftliche Online-Befragungen, zu denen registrierte Personen ab 18 Jahren auf freiwilliger Basis eingeladen werden. Die Erhebung erfolgte vom 19.06.2021 bis 13.07.2021 zwischen der dritten und vierten COVID-19-Welle [38].

Insgesamt nahmen 833 Personen an der Befragung teil. Nach Ausschluss von Personen, die nicht volljährig waren (*n* = 4) oder unplausible Antworten gaben (*n* = 1), ergab sich eine finale Stichprobe von *n* = 828. Die Teilnehmenden wurden zu Beginn umfassend aufgeklärt und um ihre informierte Einwilligung gebeten. Sie hatten bei jeder Frage die Möglichkeit, keine Angabe zu machen.

### Erhebungsinstrument und Operationalisierung

#### Häufigkeit und Inhalt der Weitergabe von Informationen zur COVID-19-Impfung

Die Häufigkeit und Inhalte der Weitergabe von Informationen zur COVID-19-Impfung wurden auf einer Skala von 1 (*nie*) bis 7 (*sehr häufig*) erfasst. Bei der Häufigkeit wurde zwischen der Weitergabe an Fremde („Wie oft teilen Sie Informationen zur COVID-19-Impfung, die auch fremde Personen erreichen, z. B. über soziale Medien oder in Messenger-Gruppen?“, Mittelwert (*M*) = 1,66; Standardabweichung (*SD*) = 1,31) und Personen im sozialen Umfeld („Wie oft teilen Sie Informationen zur COVID-19-Impfung mit ihrem sozialen Umfeld, z. B. über private Nachrichten oder persönliche Gespräche?“, *M* = 4,56; *SD* = 1,80) differenziert. Um den Inhalt zu erfassen, wurden diejenigen, die schon einmal Informationen weitergegeben haben (an Fremde: *n* = 254; an soziales Umfeld: *n* = 777), gefragt, wie oft sie Informationen zu Vorteilen der Impfung (an Fremde: *M* = 2,63; *SD* = 1,90; an soziales Umfeld: *M* = 4,36; *SD* = 1,89), zu Risiken (an Fremde: *M* = 2,45; *SD* = 1,88; an soziales Umfeld: *M* = 3,78; *SD* = 1,78) sowie zur eigenen Impferfahrung (an Fremde: *M* = 2,28; *SD* = 1,71; an soziales Umfeld: *M* = 4,14; *SD* = 2,05) weitergeben.

### Informationssuche zur COVID-19-Impfung über Online-Informationsquellen

Die Items zur Erfassung der Nutzung verschiedener Informationsquellen orientierten sich an einer vergleichbaren Abfrage des *Health Information National Trends Survey* (HINTS) Germany [38]. Die Teilnehmenden gaben auf einer Skala von 1 (*nie*) bis 7 (*sehr häufig*) an, wie häufig sie bestimmte Quellen (Tab. 1) nutzen, um sich über die Impfung zu informieren.

**Table 1:** 

Prädiktoren: Informationssuche über Informationsquellen	Modell 1 Informationen zu Vorteilen der Impfung weitergeben			Modell 2 Informationen zu Risiken der Impfung weitergeben			Modell 3 Informationen zur Impferfahrung weitergeben		
	*B*	*SE*	β	*B*	*SE*	β	*B*	*SE*	β
Tageszeitungen, politische Wochenzeitungen (inkl. Online-Angebote)	0,08	0,03	0,09*	0,08	0,03	0,10*	0,13	0,04	0,14***
Öffentlich-rechtliche und private Fernsehsender (inkl. Online-Angebote)	0,21	0,03	0,23***	0,05	0,03	0,05	0,15	0,04	0,15***
Radiosender (inkl. Online-Angebote)	0,02	0,03	0,02	0,03	0,03	0,04	0,03	0,04	0,03
Suchmaschinen	0,03	0,04	0,04	0,06	0,04	0,07	0,00	0,04	0,00
Online-Lexika	0,01	0,05	0,01	−0,03	0,04	−0,03	−0,06	0,05	−0,05
Gesundheitsportale	0,08	0,05	0,06	0,07	0,05	0,06	0,02	0,06	0,01
Ratgeber-Communitys oder spezielle Gesundheitsforen	−0,06	0,07	−0,03	0,12	0,07	0,07	0,09	0,08	0,05
Soziale Medien	0,05	0,04	0,04	0,04	0,04	0,04	0,06	0,04	0,05
Webseiten von Ärzt*innen, medizinischen Einrichtungen oder Krankenkassen	−0,02	0,05	−0,02	0,01	0,05	0,01	0,02	0,05	0,02
Webseiten von Gesundheitsbehörden (z. B. BZgA, RKI, Gesundheitsämter)	0,18	0,04	0,19***	0,01	0,04	0,01	0,21	0,04	0,21***
YouTube-Videos	−0,10	0,05	−0,08*	0,12	0,05	0,11**	−0,15	0,05	−0,11**
Messenger-Dienste	−0,13	0,05	−0,11**	0,16	0,05	0,15***	−0,13	0,05	−0,11**
Nagelkerkes* R*^*2*^ *(kor)*	0,182			0,111			0,147		
*F*	14,50***			8,56***			11,34***		

Prädiktoren der Weitergabe von Informationen zu den Vorteilen der COVID-19-Impfung (Modell 1, *n* = 728), den Risiken der COVID-19-Impfung (Modell 2, *n* = 728) und zur eigenen COVID-19-Impferfahrung (Modell 3, *n* = 723) an Personen aus dem sozialen Umfeld. Die Stichproben enthalten jeweils nur die Personen, die allgemein Informationen zur COVID-19-Impfung weitergeben. Alle Varianzinflationsfaktoren (VIF) < 5

*B* Regressionskoeffizient, *β* Beta/standardisierter Regressionskoeffizient, *F* F‑Wert des zugrunde liegenden F‑Tests, *BZgA* Bundeszentrale für gesundheitliche Aufklärung, *RKI* Robert Koch-Institut, *R*^*2*^* (kor)* korrigiertes Bestimmtheitsmaß, *SE* Standardfehler

*** *p* ≤ 0,001, ** *p* ≤ 0,01, * *p* ≤ 0,05

### Motive der Weitergabe von Informationen zur COVID-19-Impfung

Um die Motive für und gegen eine Informationsweitergabe zu identifizieren, wurden insgesamt sieben Motive auf einer Skala von 1 (*trifft ganz und gar nicht zu*) bis 7 (*trifft voll und ganz zu*) erfasst. Die Motive für eine Informationsweitergabe waren, *anderen Menschen helfen* („Es fühlt sich gut an, jemandem zu helfen, indem ich Informationen zur COVID-19-Impfung teile“, *M* = 4,25; *SD* = 1,93; [39, 40]), *sozialer Austausch* („Ich teile Informationen zur COVID-19-Impfung, um mich mit anderen Menschen auszutauschen“, *M* = 4,33; *SD* = 2,02; [11, 41]), *Aufmerksamkeit schaffen* („Ich teile Informationen zur COVID-19-Impfung, um Menschen aufzuklären“, *M* = 4,72; *SD* = 1,95; [35]) und *Statusgewinn* („Ich stehe gut da, wenn ich Informationen zur COVID-19-Impfung teile“, *M* = 2,42; *SD* = 1,58; [11, 41]). Diejenigen, die bisher nie Informationen zur Impfung weitergegeben haben, wurden hier ausgeschlossen. Als mögliche Motive gegen die Informationsweitergabe wurden in Anlehnung an Neubaum und Krämer [37] *Angst vor Ablehnung* („Ich befürchte, von anderen abgelehnt zu werden, wenn ich Informationen zur COVID-19-Impfung teile“, *M* = 2,04; *SD* = 1,63), *Angst vor Verurteilung* („Ich befürchte, einen schlechten Ruf zu bekommen, wenn ich Informationen zur COVID-19-Impfung teile“, *M* = 1,82; *SD* = 1,53) und *Angst vor Angriffen (*„Ich befürchte, von anderen beleidigt zu werden, wenn ich Informationen zur COVID-19-Impfung teile“, *M* = 2,22; *SD* = 1,80) erhoben.

### Datenanalyse

Um Assoziationen zwischen der Informationsweitergabe an Personen aus dem sozialen Umfeld bzw. Fremde und dem Alter, Geschlecht und Impfstatus zu untersuchen (F1), wurden zwei multiple lineare Regressionsanalysen durchgeführt. Deskriptive Statistiken zeigten, um welche Inhalte es sich dabei handelte (F2). Ebenfalls mithilfe von multiplen linearen Regressionsanalysen wurden der Zusammenhang zwischen der Nutzung verschiedener Informationsquellen und der Informationsweitergabe (F3) sowie mögliche Motive untersucht (F4).

## Ergebnisse

### Demografie der Stichprobe

Die Teilnehmenden waren im Alter von 18–90 Jahren (*M* = 47,78; *SD* = 15,68) und zu 55,1 % weiblich. Sie hatten überwiegend einen hohen Bildungsabschluss (2,5 % niedriger Bildungsstand; 13,6 % mittlerer Bildungsstand; 83,8 % hoher Bildungsstand). Die Mehrheit der Befragten (75,1 %) war mindestens einmal gegen COVID-19 geimpft.

### Wer gibt Informationen zur COVID-19-Impfung an wen weiter (F1)?

Insgesamt wurden Informationen zur COVID-19-Impfung häufiger an Personen aus dem sozialen Umfeld (*M* = 4,56; *SD* = 1,80) als an Fremde (*M* = 1,66; *SD* = 1,31) weitergegeben.

Die erste multiple lineare Regressionsanalyse zur Weitergabe von Informationen im sozialen Umfeld (Tab. 2, Modell 1, korrigiertes (*kor*) Nagelkerkes *R*^*2*^ = 0,019) zeigte, dass Alter (β = 0,09; *p* = 0,017) und Geschlecht (β = −0,13; *p* < 0,001) mit der Informationsweitergabe assoziiert waren. Je älter eine Person, desto häufiger wurden Informationen zur COVID-19-Impfung im sozialen Umfeld weitergegeben. Ebenso berichteten Frauen häufiger Informationen in ihrem sozialen Umfeld weiterzugeben als Männer. Der Impfstatus (β = 0,06; *p* = 0,110) war nicht mit der Informationsweitergabe im sozialen Umfeld assoziiert.

**Table 2:** 

	Modell 1 Informationsweitergabe an Personen aus dem sozialen Umfeld			Modell 2 Informationsweitergabe an fremde Personen		
	*B*	*SE*	β	*B*	*SE*	β
Alter	0,01	0,00	0,09*	0,01	0,00	0,10**
Geschlecht (Ref. weiblich)	−0,46	0,13	−0,13***	−0,06	0,09	−0,02
Impfstatus (Ref. ungeimpft)	0,24	0,15	0,06	−0,26	0,11	−0,09*
Nagelkerkes* R*^*2*^* (kor)*	0,019			0,011		
*F*	6,33***			3,94**		

Prädiktoren der Weitergabe von Informationen zur COVID-19-Impfung an Personen aus dem sozialen Umfeld (Modell 1, *n* = 818) und an fremde Personen (Modell 2, *n* = 818). Alle Varianzinflationsfaktoren (VIF) < 5

*B* Regressionskoeffizient, *β* Beta/standardisierter Regressionskoeffizient,* F* F-Wert des zugrunde liegenden F‑Tests, *R*^*2*^* (kor)* korrigiertes Bestimmtheitsmaß, *Ref.* Referenzkategorie, *SE* Standardfehler

*** *p* ≤ 0,001, ** *p* ≤ 0,01, * *p* ≤ 0,05

Die zweite multiple lineare Regressionsanalyse zur Weitergabe von Informationen an fremde Personen (Tab. 2 Modell 2, Nagelkerkes *R*^*2*^* (kor)* = 0,011) ergab, dass Alter (β = −0,13; *p* = 0,005) und Impfstatus (β = −0,13; *p* = 0,015) mit der Informationsweitergabe assoziiert waren. Je älter eine Person, desto häufiger wurden Informationen zur COVID-19-Impfung an Fremde weitergegeben. Ebenso berichteten Personen ohne COVID-19-Impfung im Vergleich zu Personen mit mindestens einer Impfung häufiger Informationen an Fremde weiterzugeben. Das Geschlecht (β = −0,13; *p* = 0,515) war nicht mit der Weitergabe von Informationen an Fremde assoziiert.

### Welche Informationen zur COVID-19-Impfung werden weitergeben (F2)?

Da sich in den vorherigen Analysen gezeigt hatte, dass die Informationsweitergabe der Teilnehmenden vor allem im sozialen Umfeld stattfand, wurde in der folgenden Analyse nur die Informationsweitergabe im sozialen Umfeld betrachtet. Am häufigsten wurden Informationen zu den Vorteilen der Impfung (*M* = 4,36; *SD* = 1,89) weitergegeben, gefolgt von Informationen zur eigenen Impferfahrung (*M* = 4,14; *SD* = 2,05) und zu Risiken (*M* = 3,78; *SD* = 1,78).

### Wie hängt die Informationssuche mit der Weitergabe von Informationen zusammen (F3)?

Die multiple lineare Regressionsanalyse zur Weitergabe von Informationen zu den Vorteilen der COVID-19-Impfung (Tab. 1, Modell 1, Nagelkerkes *R*^*2*^* (kor)* = 0,182) zeigte, dass die Nutzung von Tageszeitungen und politischen Wochenzeitungen (β = 0,08; *p* = 0,016), öffentlich-rechtlichen und privaten Fernsehsendern (β = 0,23; *p* < 0,001) und Webseiten von Gesundheitsbehörden (z. B. Bundeszentrale für gesundheitliche Aufklärung (BZgA), Robert Koch-Institut (RKI) oder Gesundheitsämter) positiv mit der Weitergabe von Informationen über Vorteile zur COVID-19-Impfung assoziiert war (β = 0,19; *p* < 0,001), während die Nutzung von Messenger-Diensten (β = −0,11; *p* = 0,007) und YouTube (β = −0,08; *p* = 0,033) negativ damit zusammenhing. Die Nutzung von Radiosendern, Suchmaschinen, Online-Lexika, Gesundheitsportalen, Ratgeber-Communitys und speziellen Gesundheitsforen, sozialen Medien und Webseiten von Ärzt*innen, medizinischen Einrichtungen oder Krankenkassen war nicht mit der Weitergabe von Informationen über Vorteile assoziiert.

Bezüglich der Weitergabe von Informationen zu den Risiken der COVID-19-Impfung zeigte eine weitere multiple lineare Regressionsanalyse (Tab. 1, Modell 2, Nagelkerkes *R*^*2*^* (kor)* = 0,111), dass die Nutzung von Tageszeitungen und politischen Wochenzeitungen (*β* = 0,10; *p* = 0,013), Messenger-Diensten (β = 0,15; *p* < 0,001) und YouTube (β = 0,11; *p* = 0,009) positiv mit der Weitergabe von Informationen über Risiken assoziiert war. Die Nutzung von öffentlich-rechtlichen und privaten Fernsehsendern, Radiosendern, Suchmaschinen, Online-Lexika, Gesundheitsportalen, Ratgeber-Communitys und speziellen Gesundheitsforen, sozialen Medien, Webseiten von Ärzt*innen, medizinischen Einrichtungen oder Krankenkassen und Webseiten von Gesundheitsbehörden war nicht mit der Weitergabe von Informationen über Risiken assoziiert.

Die multiple lineare Regressionsanalyse zur Weitergabe von Informationen zur eigenen COVID-19-Impferfahrung (Tab. 1, Modell 3, Nagelkerkes *R*^*2*^* (kor)* = 0,175) ergab, dass die Nutzung von Tageszeitungen und politischen Wochenzeitungen (β = 0,14; *p* < 0,001), öffentlich-rechtlichen und privaten Fernsehsendern (β = 0,15; *p* < 0,001) und Webseiten von Gesundheitsbehörden (β = 0,21; *p* < 0,001) positiv und die Nutzung von YouTube (β = −0,11; *p* = 0,004) und Messenger-Diensten (β = −0,11; *p* = 0,009) negativ mit der Weitergabe solcher Informationen zusammenhing. Die Nutzung von Radiosendern, Suchmaschinen, Online-Lexika, Gesundheitsportalen, Ratgeber-Communitys und speziellen Gesundheitsforen, sozialen Medien und Webseiten von Ärzt*innen, medizinischen Einrichtungen oder Krankenkassen war nicht mit der Weitergabe von Informationen zur Impferfahrung assoziiert.

### Was sind Motive der Weitergabe von Informationen zur COVID-19-Impfung (F4)?

Bei Motiven zur Informationsweitergabe wurde ebenfalls nur die Weitergabe im sozialen Umfeld betrachtet. Die multiple lineare Regression zur Weitergabe von Informationen über Vorteile der COVID-19-Impfung (Tab. 3, Modell 1, Nagelkerkes *R*^*2*^* (kor)* = 0,173) zeigte, dass die Motive, *anderen Menschen helfen* (β = 0,20; *p* < 0,001), *sozialer Austausch* (β = 0,14; *p* < 0,001), *Angst vor Angriffen* (β = 0,13; *p* = 0,018) sowie *Aufmerksamkeit schaffen* (β = 0,08; *p* = 0,041) positiv mit der Weitergabe von Informationen zu Vorteilen der COVID-19-Impfung assoziiert waren, während die *Angst vor Verurteilung* (β = −0,19; *p* = 0,002) negativ damit assoziiert war.

**Table 3:** 

Prädiktoren	Modell 1 Informationen zu Vorteilen der Impfung weitergeben			Modell 2 Informationen zu Risiken der Impfung weitergeben			Modell 3 Informationen zur Impferfahrung weitergeben		
	*B*	*SE*	β	*B*	*SE*	β	*B*	*SE*	β
Anderen Menschen helfen	0,19	0,05	0,20***	0,09	0,04	0,10*	0,16	0,05	0,15***
Sozialer Austausch	0,13	0,04	0,12***	0,16	0,03	0,18***	0,12	0,04	0,11**
Aufmerksamkeit schaffen	0,08	0,04	0,08*	0,14	0,04	0,15***	0,01	0,04	0,01
Statusgewinn	0,05	0,05	0,05	−0,03	0,04	−0,03	0,15	0,05	0,12**
Angst vor Ablehnung	−0,10	0,08	−0,09	0,00	0,08	0,00	−0,16	0,09	−0,12
Angst vor Verurteilung	−0,24	0,08	−0,19**	0,21	0,08	0,18**	−0,16	0,09	−0,12
Angst vor Angriffen	0,14	0,06	0,13*	0,00	0,06	0,00	0,13	0,07	0,11
Nagelkerkes* R*^*2*^ *(kor)*	0,173			0,123			0,122		
*F*	24,17***			16,51***			16,19***		

Prädiktoren der Weitergabe von Informationen zu den Vorteilen der COVID-19-Impfung (Modell 1, *n* = 774), den Risiken der COVID-19-Impfung (Modell 2, *n* = 774) und zur eigenen COVID-19-Impferfahrung (Modell 3, *n* = 768) an Personen aus dem sozialen Umfeld. Die Stichproben enthalten jeweils nur Personen, die allgemein Informationen zur COVID-19-Impfung weitergeben. Alle Varianzinflationsfaktoren (VIF) < 5

*B* Regressionskoeffizient, *β* Beta/standardisierter Regressionskoeffizient,* F* F-Wert des zugrunde liegenden F‑Tests, *R*^*2*^* (kor)* korrigiertes Bestimmtheitsmaß, *SE* Standardfehler

*** *p* ≤ 0,001, ** *p* ≤ 0,01, * *p* ≤ 0,05

Die Motive *sozialer Austausch* (β = 0,18; *p* < 0,001), *Angst vor Verurteilung* (β = 0,18; *p* = 0,006), *Aufmerksamkeit schaffen* (β = 0,15; *p* < 0,001) sowie *anderen Menschen helfen* (β = 0,10; *p* = 0,038) waren positiv mit der Weitergabe von Informationen zu Risiken der COVID-19-Impfung assoziiert (multiple lineare Regression, Tab. 3, Modell 2, Nagelkerkes *R*^*2*^* (kor)* = 0,123).

Bezüglich der Weitergabe von Informationen zur eigenen Impferfahrung zeigte die multiple lineare Regression (Tab. 3, Modell 3, Nagelkerkes *R*^*2*^* (kor)* = 0,122), dass die Motive, *anderen Menschen helfen* (β = 0,15; *p* = 0,001), *Statusgewinn* (β = 0,12; *p* = 0,003) und *sozialer Austausch* (β = 0,11; *p* = 0,004) positiv mit der Weitergabe von Informationen über persönliche Erfahrungen assoziiert waren.

## Diskussion

Die Ergebnisse geben Einblicke zur Weitergabe von Informationen zur COVID-19-Impfung in Deutschland. Zum Erhebungszeitpunkt dominierte die Delta-Variante mit einem Anteil von 59 % der Infektionen [42]. Mitte Juli befürworteten 83 % der deutschen Bevölkerung die Impfung gegen COVID-19 [43]. Rund 60 % der Bevölkerung hatten bereits die erste Impfung erhalten; rund 45 % hatten den vollständigen Impfschutz und es erfolgten erste Auffrischungsimpfungen (0,01 %; [44]).

Die Studie liefert aufgrund der Charakteristika des SoSci-Panels belastbare Ergebnisse für den hochgebildeten Teil der deutschen Bevölkerung. Die folgenden Interpretationen der Ergebnisse beziehen sich dementsprechend auf diesen Teil der Bevölkerung.

In Bezug auf die Frage, wer Informationen zur COVID-19-Impfung an wen weitergibt (F1), zeigte sich mit Blick auf die Empfänger*innen, dass Informationen häufiger im sozialen Umfeld und nur selten an fremde Personen weitergegeben werden. Das Thema Impfung ist während der Pandemie sehr präsent und bestimmt vermutlich Gespräche im sozialen Umfeld. Zudem handelt es sich um ein sehr sensibles und mit vielen Unsicherheiten verbundenes Thema, das somit wahrscheinlich bevorzugt im vertrauten Umfeld besprochen wird. Unser Ergebnis spiegelt demnach möglicherweise die Orientierungssuche im sozialen Umfeld wider, in dem Meinungen und Erfahrungen von vertrauten Personen ergründet werden. Dass Informationen hauptsächlich im sozialen Umfeld geteilt werden, ist besonders relevant mit Rückbezug zu den Erkenntnissen, dass Informationen aus dem sozialen Umfeld eine besonders starke Wirkung auf Meinungen und Verhaltensweisen haben [30]. Vor dem Hintergrund, dass insbesondere in den sozialen Medien eine Vielzahl an Informationen zur Impfung öffentlich weitergegeben und verbreitet wird [18], ist es jedoch überraschend, dass die Teilnehmenden nur selten Informationen an Fremde weitergeben. Möglicherweise ist einigen Befragten nicht bewusst, dass sie Informationen u. U. auch an Fremde weitergeben, wenn sie Informationen in den sozialen Medien teilen. Gleichzeitig könnte die Infodemie zur Zurückhaltung auf den sozialen Medien und somit im Austausch mit Fremden führen.

Unsere Ergebnisse mit Blick auf die weitergebenden Personen bekräftigen bisherige Erkenntnisse zur Bedeutung von Alter und Geschlecht für das gesundheitsbezogene Informationshandeln [27–29]. Konkret zeigte sich, dass ältere Personen, Frauen und ungeimpfte Personen tendenziell häufiger Informationen zur COVID-19-Impfung weitergeben. Ältere Personen könnten einen höheren Austauschbedarf haben, da sie häufig stärker von COVID-19 betroffen sind als jüngere Personen. Mit zunehmendem Alter steigt zudem das Gefühl, über die COVID-19-Impfung (sehr) gut informiert zu sein [25], was ebenfalls die häufigere Informationsweitergabe bedingen könnte.

In Hinblick auf das Geschlecht zeigen vorherige Studien, dass Frauen generell ein höheres Interesse an Gesundheitsthemen haben und z. B. häufiger das Internet zu gesundheitsbezogenen Zwecken heranziehen [38, 45]. Unsere Ergebnisse unterstreichen somit ein generelles Muster des gesundheitsbezogenen Informationshandelns, das die häufigere Informationsweitergabe von Frauen erklären kann. Diese zeigte sich jedoch nicht in Bezug auf fremde Personen, sondern nur im sozialen Umfeld, welches auch generell für den Austausch über die eigene Gesundheit präferiert wird [38].

Dass ungeimpfte Personen ebenfalls häufiger Informationen zur COVID-19-Impfung weitergeben könnte damit zusammenhängen, dass diese Personen die Impfentscheidung ggf. noch vor sich haben und somit ein höherer Informationsbedarf besteht. Eine Studie ergab diesbezüglich, dass die Weitergabe von Informationen mit einem höheren Informationsbedarf einhergeht [13]. Die häufigere Informationsweitergabe von ungeimpften Personen zeigte sich jedoch nur in Bezug auf fremde Personen. Ungeimpfte Personen könnten die Informationsweitergabe im sozialen Umfeld aufgrund von negativen Erwartungen an derartige Gespräche eher vermeiden [46] und stattdessen außerhalb ihres Umfelds nach Austauschmöglichkeiten suchen.

In Bezug auf die Frage, welche Informationen zur COVID-19-Impfung weitergegeben werden (F2), zeigte sich, dass sowohl Vorteile als auch Risiken der Impfung sowie persönliche Erfahrungen weitergegeben wurden. Dies unterstreicht noch einmal, dass die weitergegebenen Informationen zur COVID-19-Impfung verschiedene Wahrnehmungen und Perspektiven enthalten. Beziehen wir aktuelle Forschungserkenntnisse zum negativen Einfluss von Risiken [19, 32] und zum positiven Einfluss von Vorteilen [19, 25] auf die Impfbereitschaft ein, scheinen Bürger*innen mit ihrer Informationsweitergabe die Impfbereitschaft sowohl zu fördern als auch zu hemmen.

Die Analysen zum Zusammenhang der Nutzung unterschiedlicher Informationsquellen und der Informationsweitergabe (F3) ergaben, dass Personen, die sich über Webseiten von Gesundheitsbehörden informieren, häufiger Vorteile der Impfung und persönliche Erfahrungen weitergeben. Personen, die über YouTube oder Messenger-Dienste nach Informationen zur Impfung suchen, geben hingegen weniger Vorteile und häufiger Risiken weiter. Diese Muster decken sich mit bisherigen Erkenntnissen, dass insbesondere in den sozialen Medien subjektive Erlebnisberichte von Impfrisiken weitergegeben [47] und Impfmythen verbreitet werden [16, 18]. Hinzu kommt, dass im Social-Media-Bereich kaum Qualitätskontrollen stattfinden und Nutzende dort vermehrt auf falsche und irreführende Informationen stoßen, während offizielle Webseiten von Gesundheitsbehörden seriöse Aufklärung bieten (z. B. zusammengegencorona.de mit der nationalen Kommunikationskampagne *Deutschland krempelt die #ÄrmelHoch*).

Im Hinblick auf die Motive der Informationsweitergabe (F4) zeigte sich, dass *anderen Menschen helfen, sozialer Austausch* und *Aufmerksamkeit schaffen* Motive für die Weitergabe von Informationen sowohl zu Vorteilen als auch zu Risiken der COVID-19-Impfung sind. Auch mit Blick auf das Motiv *Aufmerksamkeit schaffen* scheint relevant, ob Individuen sowohl Aufmerksamkeit für Vorteile *und* Risiken der Impfung oder für Vorteile *oder* Risiken schaffen möchten. Die Angst vor Verurteilung wurde als Barriere für die Weitergabe von Informationen über Vorteile der COVID-19-Impfung und Motivator für die Weitergabe von Risiken identifiziert. Eine mögliche Erklärung liegt darin, dass das Lager der Impfskeptiker*innen als lauter und aggressiver wahrgenommen wird und Personen bei der Weitergabe von Vorteilen im Gegensatz zur Weitergabe von Risiken damit rechnen müssen, dass ihre Angst vor Verurteilung zu konkreter Verurteilung wird. Überraschend ist, dass die Angst vor Angriffen auf die eigene Person die Weitergabe von Impfvorteilen fördert. In Bezug auf die Weitergabe von persönlichen Erfahrungen zur COVID-19-Impfung zeigten sich als Motive ebenfalls das Bedürfnis, anderen Menschen zu helfen und sich sozial auszutauschen, sowie zusätzlich der Statusgewinn. Personen gehen vermutlich davon aus, dass sie anderen mit eigenen Impferfahrungen helfen können. Das Motiv des Statusgewinns deutet darauf hin, dass Individuen stolz auf ihre Erfahrungen mit der COVID-19-Impfung bzw. auf ihren Impfstatus sind. Zum Zeitpunkt der Erhebung wurde die Impfung möglicherweise als hohes Privileg wahrgenommen, da noch nicht alle impfwilligen Personen geimpft waren [43]. Interessant wäre, ob hier hauptsächlich positive Erfahrungen mit der COVID-19-Impfung weitergegeben wurden und die Personen sich so klar als Impfbefürworter*innen bzw. geimpfte Personen positionieren, um Anerkennung zu erlangen. Insgesamt scheinen die im Forschungsstand identifizierten Motive der allgemeinen Informationsweitergabe *sozialer Austausch *[11, 33], *anderen Menschen helfen* [10, 33, 34] und *Statusgewinn* [11, 36] auch als Motive der Weitergabe von Informationen zur COVID-19-Impfung zu gelten.

### Limitationen und zukünftige Ansatzpunkte

Die Studie weist Limitationen auf und bietet Ansatzpunkte für weitere Forschung. Erstens sind die über das SoSci-Panel erhobenen Daten nicht repräsentativ. Menschen mit niedrigem und mittlerem Bildungsstand sind unterrepräsentiert und sollten in zukünftigen Studien fokussiert werden. So könnten das Ausmaß der Informationsweitergabe sowie Motive und Quellennutzung je nach Bildungsstand variieren. Zweitens wurden die Inhalte nur in Bezug auf Vorteile, Risiken und eigene Erfahrungen untersucht und beruhen auf Aussagen der Teilnehmenden. Zudem können keine Aussagen über die Qualität der genutzten Informationsquellen getroffen werden. Inhaltsanalysen bieten einen Ansatzpunkt für zukünftige Forschung, um differenziertere Einblicke in die Inhalte der weitergegebenen Informationen zu erhalten und Quellen hinsichtlich ihrer Qualität zu bewerten. Drittens wurden nur ausgewählte Motive abgefragt. Der geringe Anteil der aufgeklärten Varianz (Nagelkerkes* R*^*2*^ *(kor)*) deutet darauf hin, dass weitere Prädiktoren zentral sind, die in Folgestudien identifiziert werden sollten. Zuletzt basiert die Studie auf Querschnittsdaten, die keine Kausalitätsannahmen ermöglichen. Zukünftige Forschung sollte Längsschnittdesigns anwenden, um kausale Zusammenhänge zu analysieren.

### Praktische Implikationen und Fazit

Die Weitergabe von Informationen zur COVID-19-Impfung ist mit Chancen und Risiken verbunden. Zum einen sollte der Prozess im Sinne des sozialen Austauschs gestärkt werden, zum anderen kann es somit zur Verbreitung von falschen oder irreführenden Informationen kommen. Dies verdeutlicht die Relevanz von Gesundheitskompetenz, die für einen funktionalen Umgang mit Gesundheitsinformationen gefördert werden sollte. Ebenso sollte ein öffentliches Verständnis für wissenschaftliche Informationen geschaffen und über Strategien der Wissenschaftsleugnung aufgeklärt werden [48].

Unsere Ergebnisse verdeutlichen zudem die Relevanz zielgruppenspezifischer Kommunikation. So sollte bei der öffentlichen Impfkommunikation neben kulturellen, bildungs- und altersspezifischen Unterschieden auch der Impfstatus berücksichtigt werden. Die Mehrheit der ungeimpften Personen bewertet bspw. die nationale Impfkampagne *Deutschland krempelt die #ÄrmelHoch* als (sehr) schlecht und hat wenig Vertrauen in die kommunizierenden Institutionen [25], sodass alternative Kommunikationsstrategien benötigt werden.

Zusammenfassend sollte die Informationsweitergabe zur COVID-19-Impfung mit seriösen und zielgruppenspezifischen Informationsangeboten gestärkt werden. Darüber hinaus sollte verdeutlicht werden, dass die Weitergabe von Vorteilen der Impfung die Impfbereitschaft anderer fördern kann und somit jede*r Einzelne dazu beitragen kann, der Impfmüdigkeit in Deutschland entgegenzuwirken.
